# Fourth Branchial Anomalies: Diagnosis, Treatment, and Long-Term Outcome

**DOI:** 10.3389/fsurg.2021.748351

**Published:** 2021-09-28

**Authors:** Annelien Boonen, Greet Hens, Jeroen Meulemans, Robert Hermans, Pierre Delaere, Vincent Vander Poorten

**Affiliations:** ^1^Otorhinolaryngology-Head and Neck Surgery, University Hospitals Leuven, Leuven, Belgium; ^2^Department of Oncology, Section Head and Neck Oncology, KU Leuven, Leuven, Belgium; ^3^Department of Radiology, University Hospitals Leuven, Leuven, Belgium

**Keywords:** branchial anomaly, piriform sinus, neck infection, neck abscess, thyroiditis, endoscopic repair, fourth branchial fistula

## Abstract

**Introduction:** Fourth branchial anomalies, the rarest among anomalies of the branchial apparatus, often present diagnostic and therapeutic challenges. We evaluated the clinical presentation and radiographic features, the treatment and the long-term outcome of patients in this setting.

**Patients and Methods:** Of 12 patients treated in the University Hospitals Leuven from 2004 until 2020, 12 variables were collected: date of birth, gender, age of onset of the symptoms, age at final diagnosis, presentation, laterality, previous procedures, diagnostic tools, treatment (open neck surgery, endoscopic laser excision, or combination), complications, recurrence, and period of follow-up. Descriptive statistics were calculated and results were compared to the existing literature.

**Results:** The most common clinical manifestations were recurrent neck infections with and without abcedation. Definitive diagnosis using direct laryngoscopy, visualizing the internal sinus opening, was possible in all patients. A CT study revealed the typical features of fourth branchial anomalies in seven patients out of nine, an ultrasound study in five out of nine patients. All patients underwent open neck surgery. If this was insufficient, secondary endoscopic laser resection of the ostium at the apex of the piriform sinus was performed (*n* = 4). In eight patients a thyroid lobectomy was needed for safe complete resection. Postoperative complications were minimal and at long-term, none of the patients showed further recurrence. Average time of follow-up was 8.6 years.

**Conclusions:** Direct laryngoscopy and CT are the most accurate diagnostic tools. Our recommended treatment schedule consists of complete excision of the sinus tract by open neck surgery as the primary treatment because this ensures the best results. In case of recurrence afterwards, endoscopic laser resection of the pharyngeal ostium solved the problem.

## Introduction

Anomalies of the fourth branchial pouch, first described in 1973 (1, 2), are the most uncommon of all branchial anomalies, with a prevalence of 1–4% (3–5).

Embryological development of the laterocervical region is closely linked with that of the branchial apparatus, which forms within the first months of intra-uterine life (6–9). The branchial apparatus is composed of arches, that are separated by clefts on the external side and by pouches on the internal side. Both clefts and pouches develop into the mature structures of the head and neck (10). If obliteration is incomplete, cysts, sinuses, or fistulae can arise. Distinction between various terms to describe the fourth branchial anomalies, is important. A sinus is formed when the tract is open either to the gut (pharynx) or to the skin. A cyst is formed when there is no communication to either of the two (11–13). It can however be associated with a sinus or a fistula. A branchial fistula forms when there is a remnant of both the pouch and the cleft with rupture of the membrane.

**Figure 1:**
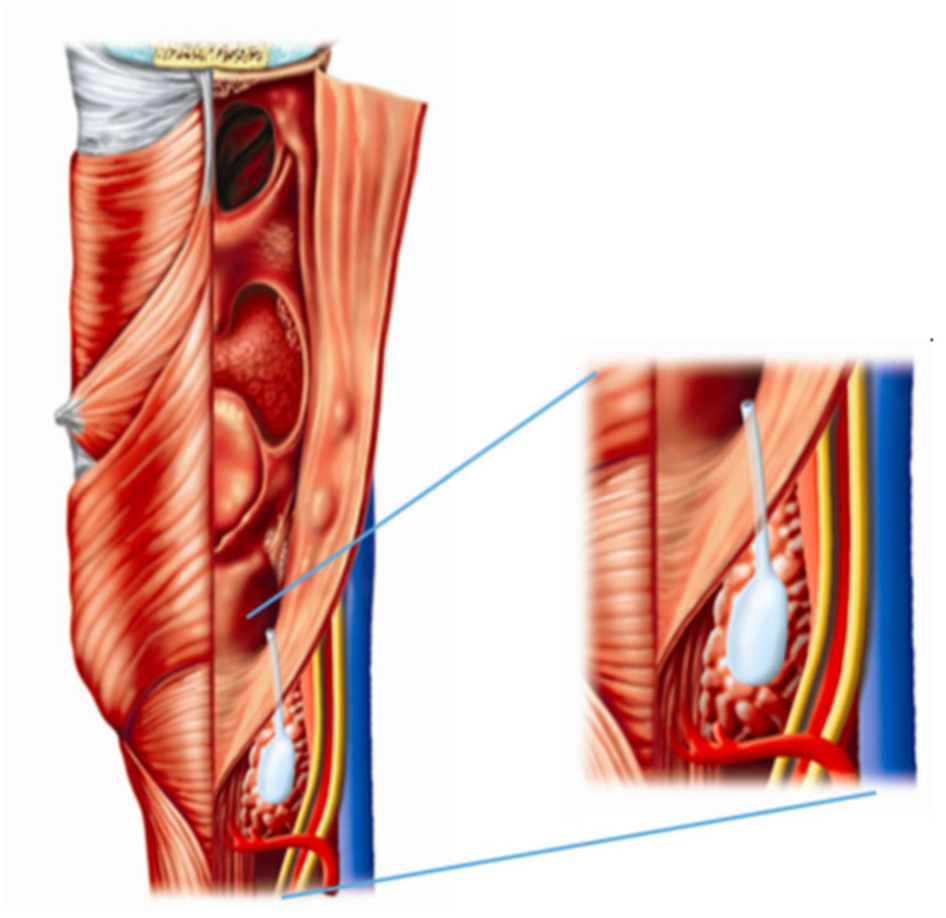
The predicted tract of a fourth branchial anomaly based on embryological development.

Fourth branchial pouch fistulas correlate with the persistence of the pharyngobranchial duct. This duct lies between the pharynx on one side, and between the upper parathyroid gland and ultimobranchial body on the other side (14, 15) (Figure 1). The ultimobranchial body gives rise to the calcitonin-secreting parafollicular cells located at the thyroid gland. A congenital fistula formed by the fusion of persistent fourth branchial pouch and cleft elements would follow a path between fourth- and sixth- arch structures (the fifth arch does not persist in man) (16). Starting from the apex of the piriform sinus, the fistula would be likely to emerge between the thyroid and cricoid cartilages, caudal to the superior laryngeal nerve, and cranial to the recurrent laryngeal nerve, which is derived from the sixth arch (9, 17, 18).

To the best of our knowledge, there has been no case reported of a complete fourth branchial fistula (19). A pseudo-fistula is possible after incision and drainage of a cervical abscess. Most frequently, a fourth branchial pouch sinus will have an internal opening in the apex of the piriform sinus and the tract perforates the cricopharyngeus muscle to end posterior to the thyroid lobe, where an abscess collection can be the presenting symptom.

Fourth branchial anomalies are located almost exclusively on the left side (16, 20, 21). The reason for this exclusive location has not been conclusively established. It has been suggested that this finding may be related to the asymmetry observed in vascular development between the left and right fourth arches (16, 20). Normal embryological development of the branchial apparatus shows that the fourth arch artery on the left side becomes part of the aortic arch during development, while the fourth arch artery on the right side becomes the proximal part of the right subclavian artery (18, 22). However, the preferential left-sided development of the ultimobranchial bodies in most mammal species, for reasons still unknown, can also be an explanation (12, 23, 24).

These anomalies often present diagnostic and therapeutic challenges. Despite findings on ultrasound, CT, barium swallow studies and endoscopic evaluation via direct laryngoscopy, diagnosing fourth branchial anomalies remains difficult in some patients. At our University center, a relatively high number of patients have presented with this condition, be it previously undiagnosed or misdiagnosed (and inadequately treated). In this study, we investigated the clinical presentation and radiographic features, the treatment, and the long-term outcome of this cohort.

## Patients and Methods

In this retrospective study, 12 consecutive subjects were enrolled, including all the patients treated at our hospital over a period of 15 years (2004–2019). Data were gathered by researching the digital files of the patients. The primary precondition to be included was a confirmed diagnosis of a fourth branchial anomaly by visualizing the internal opening of the tract at the apex of the piriform sinus. We also contacted the general practitioner of each patient to complete the information on long-term outcome (possible recurrence or late complications).

This study was performed according to the Belgian legislation after approval by the Ethics Committee of University Hospitals Leuven (IRB number: MP010465).

Twelve variables were retrieved for descriptive analysis: date of birth, gender, age of onset of the symptoms, age at final diagnosis, presentation, laterality (side), previous procedures (previous incision and drainage or previous excision), diagnostic tools (ultrasound, CT-scan, MRI, Barium swallow study, direct laryngoscopy, histopathology), treatment (open neck surgery, endoscopic laser excision, or combination), complications, recurrence, and period of follow-up.

## Results

Supplementary Table 1 summarizes the results section.

### Clinical and Radiological Features

Of all included patients, four were male and eight were female (1:2). Average follow-up time following treatment in our center was 8.6 years (ranging: 1–192 months).

The age at which patients developed symptoms and were diagnosed, varied remarkably, ranging from prenatally visible and symptomatic malformations, to the oldest patient who developed symptoms at the age of 79. The mean time between onset of the symptoms and the final diagnosis of a fourth branchial anomaly was 4.08 years, ranging from a few days to more than 25 years. Five patients were diagnosed in the same year the symptoms first occurred. The patient with the longest delay until diagnosis suffered from recurrent neck infections with abcedation for 25 years, before the underlying fourth branchial anomaly was discovered. In all patients, the malformation was the unilateral form with the sinus tract located on the left, with the exception of two patients with a rare localization on the right side of the neck.

The most common manifestation were recurrent neck infections with abcedation, which were seen in five of the twelve patients. They all required several incisions with drainage and intravenous antibiotics before their curative therapeutic surgical procedure took place (an average of 3.2 procedures preceded presentation at our institution). Three patients presented with recurrent neck infections without abscess formation. A painless swelling of the neck without inflammatory signs was seen in three other patients, who were all under 8 years of age. One of them was a newborn of 3 days old, and showed inspiratory stridor, mostly during crying, because of the mass compressing the trachea. It was also observed that during crying and being fed, the mass located in the neck increased in volume, filling with air or milk. Finally, one patient presented with an episode of suppurative thyroiditis.

**Figure 2:**
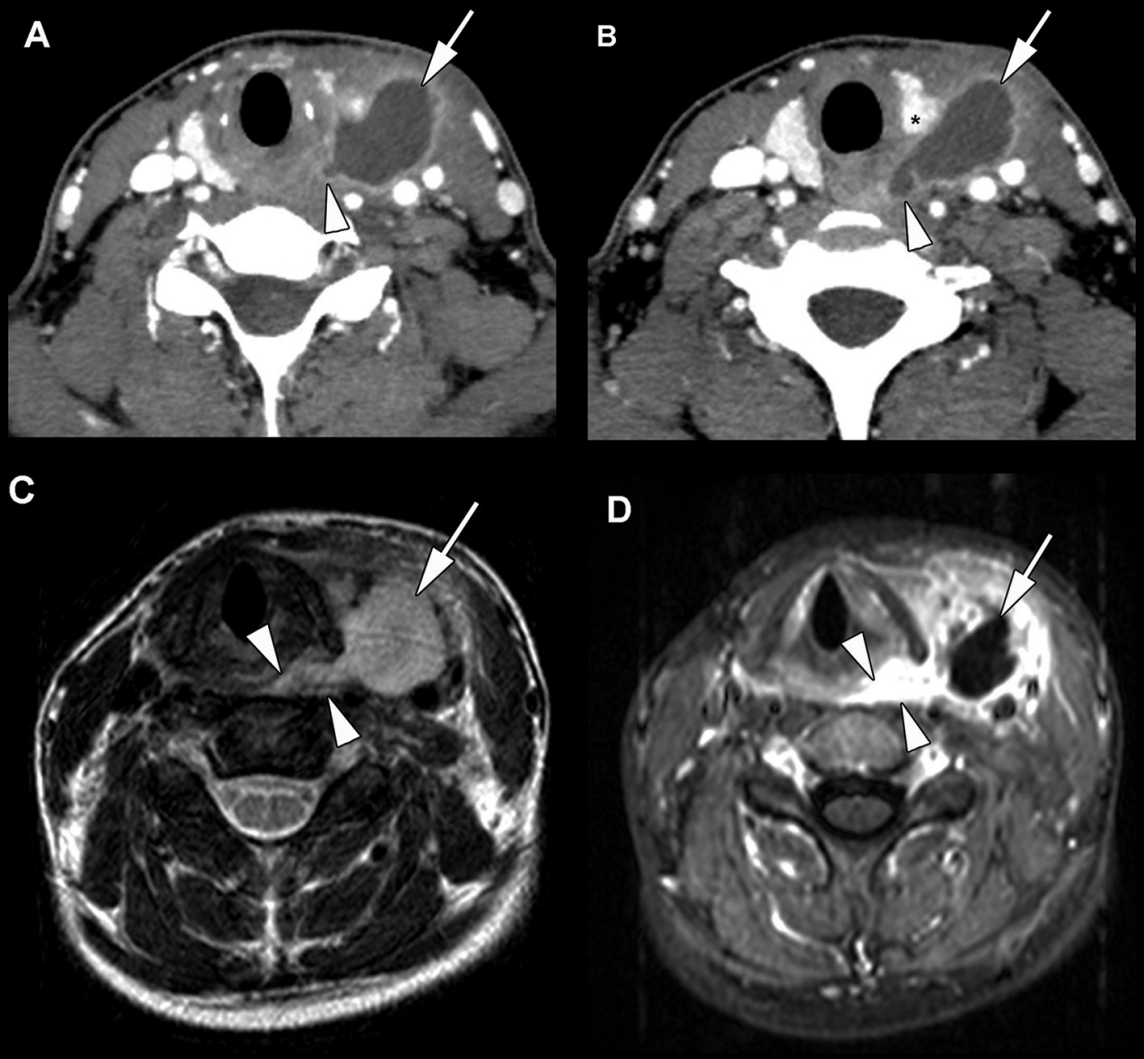
Nineteen year old patient, presenting in the emergency department with fever, throat pain, and left side neck swelling. The patient experienced three similar episodes during the last 3 years. **(A,B)** Contrast-enhanced CT study, obtained at admission to the emergency department. Hypodense mass lesion with rim enhancement (arrow) between the left thyroid lobe (X) and neck vessels, extending to the region of the left pyriform sinus apex and esophageal verge (arrowhead). These findings are compatible with an abscess secondary to a fourth branchial arch fistula. After abscess drainage and intravenous antibiotics, direct laryngoscopy confirmed the presence of a fistula originating from the left pyriform sinus. After endoscopic resection of the fistula, the patient remained symptom free. **(C,D)** MRI study, performed in another hospital, 3 years earlier. T2-weighted **(C)** and gadolinium-enhanced T1-weighted **(D)** image show similar findings as in images **(A,B)** collection (arrow) with surrounding inflammation, connecting to the pyriform sinus (arrowheads). This was recognized as a neck abscess, but the diagnosis of a fourth branchial arch anomaly was not made. Treatment at that time was abscess drainage and intravenous antibiotics.

Various diagnostic tools were used in order to establish the presence of a fourth branchial anomaly. Definitive diagnosis can be made if the orifice of the sinus tract is located at the apex of the ipsilateral sinus piriformis. In all patients, this important feature was visible using direct laryngoscopy. In one patient, this was the only diagnostic tool indicating that the symptoms originated from a fourth branchial anomaly. An ultrasound of the neck was done in nine patients out of twelve. Five of these showed the possible trajectory of a fourth branchial arch remnant. CT-studies were performed in nine patients, all showed inflammation and abcedation within the thyroid and perithyroidal tissues, which were interpreted as specific features of fourth branchial arch remnants in seven patients (Figures 2A,B). An MR study was done in seven patients. Although showing signs of inflammation and abcedation within the thyroid and perithyroidal tissues, this was prospectively never interpreted as characteristics suggesting a fourth branchial anomaly. However, in retrospect, the MR studies of four of these patients did show suggestive or typical changes of a fourth branchial pouch anomaly (Figures 2C,D). Six patients had a barium swallow study. The origin and course of the fistula, beginning at the apex of the piriform sinus, were seen by this examination in only two patients.

### Treatment

All patients, of which five underwent repeated abscess drainages in the referring centers, had open neck surgery at our medical center. One patient already underwent an open excision via a Sistrunk procedure at the referring center because the symptoms were mistakenly assumed to be originating from a thyroglossal duct cyst. In case of active infection on presentation, patients were first treated with intravenous antibiotics, or if the patient presented with an abscess collection (*n* = 5), additional incision and drainage of a residual abscess was carried out. After the infection had cooled down for at least 6 weeks, we proceeded with surgery.

**Figure 3:**
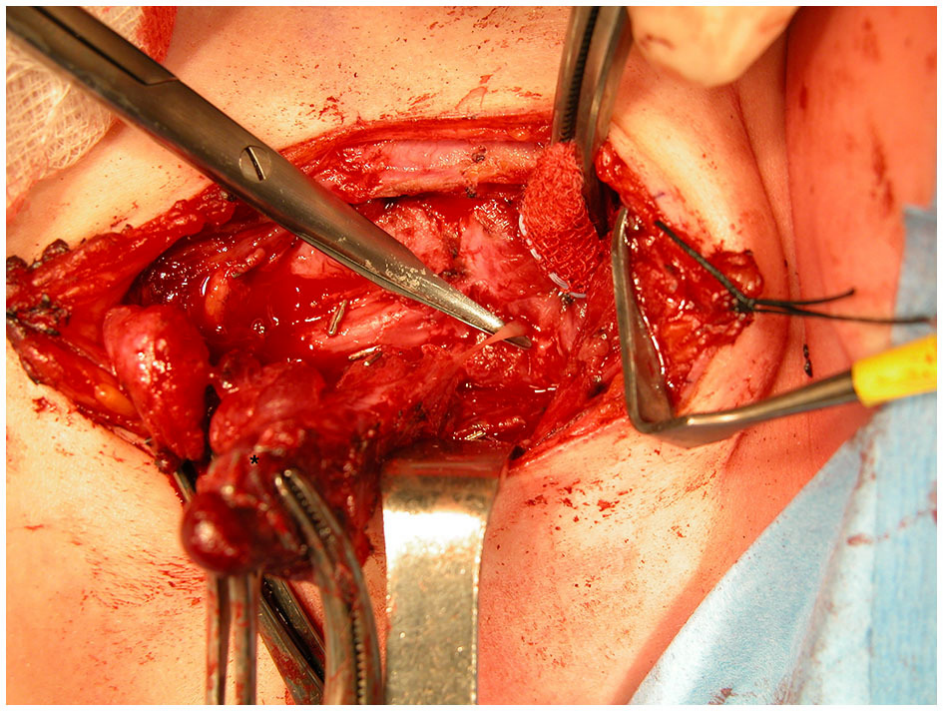
Visualizing the tract of the sinus. Clamps are holding the involved left thyroid lobe (asterisk). Arrowhead indicates the spared left recurrent laryngeal nerve.

In all 12 patients, we carried out an open resection of the fourth branchial anomaly aiming at complete excision of the sinus tract in continuity with any associated masses up to and including a few millimeters of tract inside the inferior pharyngeal constrictor (Figure 3). When dissecting the thyroid lobe, the parathyroid glands have to be carefully preserved; the recurrent laryngeal nerve also has to be identified and a sufficient portion should be dissected to ensure the nerve remains intact. The part of the inflamed thyroid lobe that is in continuity with the tract of the fistula or cyst has to be removed. As such eight of our patients needed a thyroid lobectomy (one patient who only needed a partial lobectomy after identification and preservation of the recurrent laryngeal nerve). One of these procedures was executed with the use of recurrent laryngeal nerve monitoring (Supplementary Table 1, Patient number 9).

After mobilizing the thyroid, the tract of the sinus can be identified and followed through the pharyngeal constrictor muscles. Ligation of the tract needs to be done as close to the internal orifice as possible.

The patient who underwent a Sistrunk procedure, was referred to us after the symptoms reappeared after surgery. This patient underwent first an open exploration because there was suspicion of a remnant of the previously diagnosed thyroglossal duct cyst. However, during the external approach, dissection of the inflammatory process revealed a tract running toward the apex of the right piriform sinus, which was ligated at the entrance through the pharyngeal constrictor. We then decided to perform a direct laryngoscopy to confirm a fourth pouch origin, and indeed, the internal ostium at the apex of the piriform sinus was visualized. When infections with abcedation recurred after the open approach, we hypothesized the origin of the remaining problem had to be the remaining tract of a few millimeters, internally to the pharyngeal constrictor, that cannot be removed via the external approach. Following this hypothesis, the internal ostium as well as a few millimeters of mucosa-lined tract running through the pharyngeal constrictor, up to where the external approach had ended, was subsequently resected by endoscopic carbon dioxide laser resection This patient has now remained free of disease for 24 months. Three other patients additionally needed a secondary endoscopic laser excision of the internal ostium, because symptoms reappeared.

Postoperative complications were minimal. Vocal fold function remained normal in all patients. Thyroid function was routinely checked in the patients that had undergone thyroid tissue resection; one patient showed a temporary asymptomatic hypothyroidism after a left thyroid lobectomy. This was treated with thyroid hormone replacement therapy and could be discontinued after 1 year, when thyroid function of the remaining lobe proved to be sufficient.

To the best of our knowledge, this series is also the first to report a prenatally suspected diagnosis of a fourth branchial anomaly. This condition was visualized prenatally by ultrasound, and because of its considerable volume, labor was induced. After birth, a prominent mass was located at the left side of the neck, however without effect on the respiratory system. Three weeks postnatally, because of increasing volume upon feeding, the cyst was removed surgically, as was the whole fistula tract and a part of the left lobe of the thyroid gland.

Follow-up completion by contacting the general practitioner was possible for all but one patient (no GP registered in the medical file; for this patient we used the information of the last check-up appointment at our hospital; Supplementary Table 1, Patient number 10). This made the average period of follow-up 8.6 years (range: 1–192 months). During this period, none of the 12 patients showed recurrence after undergoing our treatment schedule (primary open neck surgery, with subsequently endoscopic laser resection in case of reoccurring symptoms). There were also no late complications reported.

After the patients underwent surgery, the surgical specimen was examined in order to verify if the histopathological features matched the diagnosis of a branchial remnant. Generally, the fibrous wall of the cystic cavity was lined by stratified squamous epithelium but could also be pseudostratified, columnar ciliated epithelium in some patients (25). The connective tissue lying underneath contained abundant lymphoid tissue with multiple germinal centers. In all of our patients, histopathological samples showed these typical characteristics.

## Discussion

Fourth branchial anomalies may present in a variety of ways. The majority of our patients presented with recurrent neck infections, with or without abcedation, located at the neck along the anterior border of the sternocleidomastoid muscle. These infections recurred after antibiotic or surgical incision and drainage. Other manifestations were painless uninfected neck masses and suppurative thyroiditis. Of note, symptoms of a fourth branchial anomaly do not always occur during the first and second decades of life, as is often suggested by current literature (26, 27). This case series shows that fourth branchial remnants are not an exclusively pediatric condition, but are a very plausible explanation for recurrent neck infections and other symptoms, as already mentioned above, in adults and even the elderly.

Multiple diagnostic studies have already been proposed as the golden standard in order to establish the diagnosis of fourth branchial pouch sinuses. Our experience showed that direct laryngoscopy is the gold standard test in targeting the apex of the piriform sinus as the origin of the tract, which is an imperative characteristic. In our series, a direct laryngoscopy was executed and could visualize the ostium of the sinus in every patient. A barium swallow study could also demonstrate the tract itself in theory (28–31), but based on our data, this test was often less sensitive because the tract rarely fills with contrast agent, probably due to inflammatory soft tissue swelling or scarification. Therefore, we do not recommend this as a primary tool for diagnosing fourth branchial anomalies, but it might be useful if both ultrasound and CT-studies are negative and there remains clinical suspicion.

CT however, was the radiological examination of our choice. It proved very helpful in identifying the tract and the relationship with the surrounding structures, in particular with the thyroid gland (14, 32). In seven out of the nine patients who had a CT-study, it led us to the definitive diagnosis, demonstrating intrathyroidal cellulitis, soft tissues inflammation, or abcedation, which are all typical for a fourth branchial remnant. CT was also superior in detecting air in the sinus or fistulous tract and in evaluating thyroid involvement (14, 28, 33).

An ultrasound study of the neck may also be helpful: it can demonstrate cellulitis of the skin and the subcutaneous tissues and an irregular outline of the thyroid gland or an abscess at the exact place where the thyroid lobe would be expected. In five out of nine patients, ultrasound showed the possible trajectory of a fourth branchial arch remnant. Taking into account that a lot of the patients are children and young adults (34), the benefits of an ultrasound (no exposure to radiation) do make it a very good alternative to CT. In this case series, MRI appeared less useful to diagnose this condition, although retrospectively in many patients the characteristics of a fourth branchial remnant could be recognized. Radiologists may not be familiar with the imaging findings in this relatively rare entity. The presence of a neck abcess in the perithyroidal tissues, or within the thyroid lobe, especially on the left side, should always promp the search for a possible connection with the adjacent piriform sinus, allowing the correct diagnosis to be made (35).

Confirmation of the diagnosis is made at the time of neck exploration, when the tract follows the predicted tract based on embryological development, starting from the apex of the piriform sinus and passing caudally to the superior laryngeal nerve and cranially to the recurrent laryngeal nerve (9, 17, 18).

Based on our experience, we recommend an as complete as possible excision of the sinus tract by open neck surgery. The excision of the anomaly must be in continuity with any associated masses, and after resolution of the acute infection with antibiotics and incision and drainage of the infected mass if there is one. The sinus can be difficult to identify because of scar tissue, and can be intimately associated with the recurrent laryngeal nerve, which makes the operation sometimes challenging. Preserving the recurrent laryngeal nerve is of utmost importance, and often the safest way to do so in a heavily scarred area due to repeated infections and surgical drainages, is performing a thyroid lobectomy (6, 14, 33, 36). In some patients, like the one who presented with suppurative thyroiditis, the left lobe was infected and partially fused with the branchial cyst, making removal necessary.

Some studies propose an “endoscopic-only” approach to this problem. One study proposes endoscopic chemocauterization of the internal opening of the sinus tract with trichloroacetic acid as an alternative to open neck surgery (37). However, 22% of these patients suffered from recurring neck abscesses afterwards and needed further external resection. Also, when using TCA, there is a risk that the substance leaks into the esophagus, which can cause strictures (38). Several other methods for cauterization have been reported, but so far there is no agreement about which technique is superior. Derks et al. conducted a systematic review about the potential benefits of endoscopic electrocauterization in patients with third and fourth branchial pouch sinuses vs. surgery (39). They concluded that the effectiveness and recurrence rate was comparable between surgical and endoscopic treatment. However, this study had limitations like the small number of studies analyzed, with a low number of patients receiving surgical treatment. The mean period of follow-up was also relatively short or missing. This meta-analysis also included third branchial anomalies besides fourth branchial ones. Our endoscopic technique of choice, microsurgical laser resection of the internal ostium, was also not included in the minimally invasive options.

In our experience, most patients are cured after an external approach that ends its resection inside the pharyngeal constrictor fibers. Endoscopic laser resection of the ostium and the few millimeters of tract at the inside of the pharynx should only be considered in the few patients where symptoms persist after open neck surgery. In this series this was only needed in a minority of patients (*n* = 4; 33%). Our reasoning to prefer initial external resection is based on the embryologically determined anatomical course of fourth branchial remnants, where an external approach has the best chance of removing the maximum of the mucosally lined sinus tract, i.e., the part that runs laterally from the thyroid cartilage. One could consider only removing the internal sinus ostium by laser microsurgery, in analogy to the studies only addressing the internal ostium by (chemo-)cauterization, but this will leave the neck part of the mucosally lined sinus tract, unavoidably leading to recurrence, analogous to a thyroglossal duct cyst that will reappear if not excised completely (40). One could argue to combine the external resection with internal laser microsurgical resection, but this would imply a (temporary) opening from the pharynx to the (dissected) neck, potentially inducing postoperative healing problems, including pharyngocutaneous fistula formation.

Whereas, the currently proposed treatment strategy works well in our hands, the number of patients in our series remains relatively small and the study design is retrospective. Ideally a prospective study comparing treatment options (open or endoscopic, or a combination) with a substantial follow-up, could help in determining the most effective and safe treatment strategy. This study setup, to date, however, has not been performed, and remains unlikely to be performed in the future, given the rarity of fourth pouch anomalies.

## Conclusions

Fourth branchial pouch sinuses do not only present in neonates and young children, but should also be considered in older children and adults. Indeed, the condition can be asymptomatic for years, before the typical presentation of recurrent neck infections and abscesses appears. CT, ultrasound, and direct laryngoscopy are the diagnostic tools of choice and provide accurate information regarding the anomaly. MRI is also valuable seeing as in retrospect, four out of seven MRI scans clearly showed characteristics of a fourth branchial anomaly. Based on our long-term outcome data, we are in favor of performing open neck surgery as the first and best treatment option. Only if symptoms reoccur, endoscopic laser resection of the ostium and the pharyngeal part of the tract can be subsequently and safely executed.

Fourth branchial anomalies are rare, but should be considered as differential diagnosis in patients -of all ages- presenting with recurrent neck infections, since earlier recognition will lead to less futile surgical drainages, reduce the number of hospitalizations for antibiotherapy, as well as reducing potential complications when definitive resection is performed. In terms of diagnosis, imaging plats a very important role. Based on the finding that retrospecively MRI did show typical characteristics of a fourth branchial anomaly, it can be concluded that it is necessary that this condition should be included in the diferential diagnosis when analyzing imaging of patients where there is clinical suspicion.

## Data Availability Statement

The original contributions presented in the study are included in the article/Supplementary Material, further inquiries can be directed to the corresponding author/s.

## Ethics Statement

The studies involving human participants were reviewed and approved by The Research Ethics Committee UZ/KU Leuven. Written informed consent from the participants' legal guardian/next of kin was not required to participate in this study in accordance with the national legislation and the institutional requirements.

## Author Contributions

AB and VV contributed to study set-up, data collection, data quality control, data analysis (statistics), drafting manuscript, and review of manuscript. GH, JM, RH, and PD contributed to drafting manuscript and review of manuscript. All authors contributed to the article and approved the submitted version.

## Conflict of Interest

The authors declare that the research was conducted in the absence of any commercial or financial relationships that could be construed as a potential conflict of interest.

## Publisher's Note

All claims expressed in this article are solely those of the authors and do not necessarily represent those of their affiliated organizations, or those of the publisher, the editors and the reviewers. Any product that may be evaluated in this article, or claim that may be made by its manufacturer, is not guaranteed or endorsed by the publisher.
