# Supplementary material for: Fourth Branchial Anomalies: Diagnosis, Treatment, and Long-Term Outcome
Source: Front Surg. 2021 Sep 28;8:748351. doi: 10.3389/fsurg.2021.748351 (PMC8505890; doi:10.3389/fsurg.2021.748351)
Supplement: Supplementary file 1 [file Table_1.DOCX]

| Case number |  | 1 | 2 | 3 |
| --- | --- | --- | --- | --- |
| Age of onset of the symptoms (years) |  | 3 | 2 | 10 |
| Age of final diagnosis (years) |  | 3 | 3 | 26 |
| Presentation |  | neck abscess | recurrent neck abscedation | recurrent  neck infections |
| Laterality |  | left | right | left |
| Previous procedure(s) |  |  |  |  |
|  | *Previous drainage* | 2 | 3 | / |
|  | *Previous excision* | 0 | 3 | / |
| Diagnostic tools |  |  |  |  |
|  | *Ultrasound* | X: adenitis colli | X: neck abscess | / |
|  | *CT-scan* | X: suspicion of  4^th^ branchial fistula | X: suspicion of  4^th^ branchial fistula | X: visualisation  trajectory |
|  | *MRI* | / | X: suspicion remainder  ductus thyroglossus cyst | / |
|  | *Barium swallow study* | / | / | / |
|  | *Direct laryngoscopy* | X: visualisation  trajectory | X: visualisation  trajectory | X: visualisation  trajectory |
|  | *Histopathology* | X: branchial remainder | X: branchial remainder | X: branchial remainder |
| Treatment |  |  |  |  |
|  | *Open neck surgery* | X | X | X |
|  | *Endoscopic laser resection* | X | X |  |
|  | *(Hemi)thyroidectomy* | left hemithyroidectomy | / | left  hemithyroidectomy |
| Complications |  | / | / | / |
| Recurrence |  | / | / | / |
| Follow-up (years) |  | 9 | 2 | 16 |

| Case number |  | 4 | 5 | 6 |
| --- | --- | --- | --- | --- |
| Age of onset of the symptoms (years) |  | 7 | 57 | birth |
| Age of final diagnosis (years) |  | 32 | 57 | 0 (3 days) |
| Presentation |  | recurrent neck infections, abscedation | recurrent neck  infections | - swelling neck  - stridor during crying |
| Laterality |  | left | left | right |
| Previous procedure(s) |  |  |  |  |
|  | *Previous drainage* | multiple | / | / |
|  | *Previous excision* | multiple | / | / |
| Diagnostic tools |  |  |  |  |
|  | *Ultrasound* | / | X: suspicion of  4^th^ branchial fistula | X: suspicion of  4^th^ branchial fistula |
|  | *CT-scan* | X: no visualisation  trajectory | X: visualisation trajectory | / |
|  | *MRI* | / | / | X: cystic mass |
|  | *Barium swallow study* | X: visualisation trajectory | / | X: no visualisation  of trajectory to the pharynx |
|  | *Direct laryngoscopy* | X: visualisation trajectory | X: visualisation trajectory | X: visualisation trajectory |
|  | *Histopathology* | X: branchial remainder | X: branchial remainder | X: branchial remainder |
| Treatment |  |  |  |  |
|  | *Open neck surgery* | X | X | X |
|  | *Endoscopic laser resection* | X |  |  |
|  | *(Hemi)thyroidectomy* | / | left hemithyroidectomy | right hemithyroidectomy |
| Complications |  | / | / | / |
| Recurrence |  | / | / | / |
| Follow-up (years) |  | 15 | 10 | 13 |

| Case number |  | 7 | 8 | 9 |
| --- | --- | --- | --- | --- |
| Age of onset of the symptoms (years) |  | 6 | 13 | 17 |
| Age of final diagnosis (years) |  | 13 | 13 | 19 |
| Presentation |  | - adenopathy  - infection neck | suppurative thyroiditis | recurrent neck infections  with abscedation |
| Laterality |  | left | left | left |
| Previous procedure(s) |  |  |  |  |
|  | *Previous drainage* | / | / | 2 |
|  | *Previous excision* | / | / | / |
| Diagnostic tools |  |  |  |  |
|  | *Ultrasound* | X: nodulary structure | / | X: suspicion  of 4^th^ branchial fistula |
|  | *CT-scan* | X: suspicion  of 4^th^ branchial fistula | X: abscess thyroid gland | / |
|  | *MRI* | X: lymphnode package | / | X: cystic mass and  abscess neck |
|  | *Barium swallow study* | X: no visualisation  trajectory | X: visualisation  trajectory | / |
|  | *Direct laryngoscopy* | X: visualisation  trajectory | X: visualisation  trajectory | X: visualisation  trajectory |
|  | *Histopathology* | X: branchial remainder | X: branchial remainder | X: branchial remainder |
| Treatment |  |  |  |  |
|  | *Open neck surgery* | X | X | X |
|  | *Endoscopic laser resection* |  |  |  |
|  | *(Hemi)thyroidectomy* | left hemithyroidectomy | left hemithyroidectomy | left hemithyroidectomy |
| Complications |  | hypothyroidism | / | / |
| Recurrence |  | / | / | / |
| Follow-up (years) |  | 12 | 13 | 3 |

| Case number |  | 10 | 11 | 12 |
| --- | --- | --- | --- | --- |
| Age of onset of the symptoms (years) |  | 7 | prenatal | 79 |
| Age of final diagnosis (years) |  | 7 | prenatal | 81 |
| Presentation |  | painless stern swelling neck | swelling neck: cystic mass | neck abscess +  oesophagus tear |
| Laterality |  | left | left | left |
| Previous procedure(s) |  |  |  |  |
|  | *Previous drainage* | / | / | 2 |
|  | *Previous excision* | / | / | 5 |
| Diagnostic tools |  |  |  |  |
|  | *Ultrasound* | X: suspicion 4^th^ branchial fistula | X: suspicion 4^th^ branchial cyst | X: abscess neck |
|  | *CT-scan* | X: infectious process/branchial cyst | / | X: no visualisation  trajectory (3) |
|  | *MRI* | X: no distinction possible with tumoral process | X: cystic mass | X: fistula between  hypopharynx and skin |
|  | *Barium swallow study* | X: no visualisation trajectory | / | X: no visualisation  trajectory (5) |
|  | *Direct laryngoscopy* | X: visualisation  trajectory | X: visualisation  trajectory | X: visualisation  trajectory |
|  | *Histopathology* | X: branchial remainder | X: branchial remainder | X: branchial remainder |
| Treatment |  |  |  |  |
|  | *Open neck surgery* | X | X | X |
|  | *Endoscopic laser resection* |  |  | X |
|  | *(Hemi)thyroidectomy* | left hemithyroidectomy | partial left hemithyroidectomy | / |
| Complications |  | / | / | / |
| Recurrence |  | / | / | / |
| Follow-up (years) |  | 0 (1 month) | 9 | 1 |

*Table 1.* **M**: male*;* **F**: female*;* **CT**: Computed Tomography scan*;* **MRI**: Magnetic Resonance Imaging*;* **X**: executed/present in this case*;* **/**: not executed/not present in this case; **(3) – (5):** number of times the examination was performed*;* **follow-up (years**): starting at the year the therapeutic surgery at our hospital took place until 2020 -the year this study was published- or until the last check-up appointment at our hospital for the patient who did not have a general practitioner registered (patient number 10).
